# Atrial Septal Defects: From Embryology to Pediatric Pulmonary Hypertension

**DOI:** 10.3390/jcm14217698

**Published:** 2025-10-30

**Authors:** Elżbieta Bartoszewska, Anna Chrapkowska, Oliwia Zielińska, Maria Mordalska, Julia Lizon, Zuzanna Zalewska, Marek Wasicionek

**Affiliations:** 1 Faculty of Medicine, Wroclaw Medical University, Mikulicza-Radeckiego 5, 50-345 Wrocław, Poland; anna.chrapkowska@student.umw.edu.pl (A.C.); oliwia.zielinska@student.umw.edu.pl (O.Z.); maria.mordalska@student.umw.edu.pl (M.M.); julia.lizon@student.umw.edu.pl (J.L.); zuzanna.zalewska@student.umw.edu.pl (Z.Z.); 2 Students’ Scientific Group of Pediatric Cardiology, 1st Department and Clinic of Pediatric, Allergology and Cardiology, Wroclaw Medical University, Chałubińskiego 2a, 50-368 Wrocław, Poland; 3 1st Department and Clinic of Pediatric, Allergology and Cardiology, Wroclaw Medical University, Chałubińskiego 2a, 50-368 Wrocław, Poland; marek.wasicionek@umw.edu.pl

**Keywords:** atrial septal defect, pulmonary hypertension, pulmonary arterial hypertension, congenital heart defects, pediatric cardiology, Eisenmenger syndrome

## Abstract

Atrial septal defect (ASD) is characterized by an abnormal opening between the left (LA) and right atria (RA). Even though it’s one of the most prevalent congenital heart defects, there are still many knowledge gaps and clinical uncertainties. This review aims to create a complex description of ASD and discuss its link with pulmonary arterial hypertension (PAH). An extensive literature search was conducted on sites such as PubMed, Google Scholar, and ScienceDirect. This study reviews the key findings of peer-reviewed articles with the keywords ASD, PAH, and congenital heart defects. The research showed that whilst there are multiple reviews, there is still a need for a deeper understanding, especially in areas of embryology, decision thresholds for closure, and pediatric-specific long-term outcomes. Current guidelines often extrapolate data only from adults or avoid certain topics. Those ambiguities often lead to variable medical practices, missed opportunities, and uncertainty for families. This review is divided into clear sections, creating a step-by-step characterization of the most important information, which can be useful to specialists. It touches on important subjects and controversies. It shows a clear timeline, starting with embryology, genetics, and anatomy, through pathophysiology and patient description, ending with recommendations and indications for specific treatment methods. Moreover, it creates a clear connection between ASD and PAH, outlining its danger and the need for prevention.

## 1. Introduction

Congenital heart disease is a group of conditions that are present at birth, characterized by structural abnormalities. Atrial septal defect (ASD) makes up 10–15% of all of these conditions and is the second most common congenital heart disease, presenting during childhood [1]. The incidence of ASD is estimated at 2 per 1000 live births [2]. Patients with small ASD are often asymptomatic; they are not diagnosed until adulthood [3].

Small ASDs, typically defined as defects measuring less than 5 mm, are often asymptomatic [1,4,5]. These smaller defects have the potential to close spontaneously. In contrast, large ASDs—those exceeding 10 mm—are associated with significantly greater clinical risk [4]. A large atrial septal defect can present with arrhythmias (caused by atrial enlargement, which develops with time), increased incidence of pneumonia, exercise intolerance, growth retardation, increased mortality, and pulmonary hypertension [1,5].

The pathophysiological hallmark of ASD involves a left-to-right shunt, attributable to the higher pressures in the left, resulting in RV volume overload, increased pulmonary circulation, and, finally, enlargement of the right side of the heart. Over time, this can culminate in right-sided heart failure [6,7]. Without any treatment, pulmonary hypertension (PH) can develop. PH is diagnosed if PAP (pulmonary artery pressure) is higher than 25 mm Hg [8]. In this case, reversal of the shunt is possible. Referring to the right-to-left shunt, oxygenated blood mixes with deoxygenated blood, a clinical manifestation known as Eisenmenger syndrome, which can occur in cyanosis and systemic hypoxemia [1,5,9]. It usually develops over many years, and it only affects a few percent of people [9]. Also, patients with large ASD are predisposed to thromboembolic events. In such a case, an embolus can go through the atrial septal defect into the left side of the heart and generate an arterial embolic event instead of a pulmonary embolic event. It can lead to stroke, myocardial infarction (MI), and organ ischaemia [7].

Although the relationship between atrial septal defect (ASD) and the development of pulmonary arterial hypertension (PAH) has long been recognized, the literature still contains significant gaps and areas of clinical uncertainty. Most available studies focus on the basic pathophysiology and classification of ASD, while much less attention is given to the heterogeneity of its clinical course and the factors influencing the development of PAH.

ASD is one of the most common congenital heart defects; however, its etiology remains incompletely understood. While the majority of cases occur sporadically, there is growing evidence highlighting the important role of genetic and environmental factors that increase the risk of ASD, such as chromosomal syndromes (Down, Noonan, Holt–Oram, Ellis–Van Creveld), prematurity, maternal diseases (e.g., diabetes), or prenatal exposure to teratogens [10]. Nevertheless, comprehensive studies elucidating the mechanisms of interaction between these factors and their impact on ASD development are lacking.

Another unresolved issue is the optimal timing of surgical intervention. Current guidelines recommend closure of ASD between 2 and 5 years of age, but the decision must be individualized, as earlier intervention may be required in cases of right ventricular enlargement or large defects [11]. Particularly challenging are cases involving premature infants, children with Down syndrome, or those with chronic lung diseases such as bronchopulmonary dysplasia, in whom spontaneous closure is less likely and the risk of progression to PAH is higher, suggesting that earlier surgery may be necessary [12,13]. There are still no clear criteria to precisely determine when earlier intervention should be undertaken in specific patient groups [14,15].

Studies clearly demonstrate that the presence of ASD is associated with increased mortality, which rises with patient age, a trend influenced by the frequent absence of symptoms in the first decades of life [16].

In cases where Eisenmenger syndrome develops, surgical closure is contraindicated, and treatment is primarily symptomatic. Currently available targeted therapies improve outcomes, but survival rates remain unsatisfactory [17,18]. Furthermore, there is a lack of large, prospective studies assessing the efficacy and safety of these therapies in pediatric and young adult populations.

This paper was meant to create a clear characterization of ASD and PAH, displaying important information from areas of both scientific knowledge, such as genetics, embryology, anatomy, and physiology, and clinical observations, such as patient profile and treatment methods. Our aim is to highlight the urgent need for further research to address these gaps and clinical uncertainties, to refine treatment strategies tailored to specific patient subgroups, and to expand knowledge about the role of various factors in ASD development. Such efforts may help facilitate earlier diagnosis, enable timely medical intervention, and reduce the risk of long-term cardiopulmonary complications.

## 2. Embryology and Genetic Predispositions to ASD

### 2.1. ASD Development

The interatrial septum is a fibromuscular wall between the right and left atria of the heart. Its anatomical complexity reflects its distinct functional roles during fetal development and postnatal life. The development of the atrial septum begins at 4 to 5 weeks of gestation [19]. Initially, the heart first develops the septum primum, which grows downward, from the roof of the common atrium, creating two separate chambers by fusing with the endocardial cushion and closing ostium primum completely; meanwhile, another opening appears in the upper area: ostium secundum. Subsequently, the septum secundum forms to the right of the septum primum and partially overlaps the ostium secundum, leaving a residual opening termed the foramen ovale [19,20,21].

Due to this structure of the fetal heart, in circulation, oxygenated blood from the umbilical vein flows into the IVC and the right atrium of the heart. The Eustachian valve directs the blood flow toward and through the foramen ovale (later creating fossa ovalis, the thinnest part of the interatrial septum), as well as through the secondary foramen from the right atrium to the left atrium, left ventricle, and subsequently to the systemic circulation via the aorta. A portion of this blood returns to the placenta through the umbilical arteries. Simultaneously, blood comes back from the SVC and coronary arteries to the right atrium, then to the right ventricle. Most of the blood that leaves the right ventricle bypasses the non-functioning lungs through the ductus arteriosus to the descending aorta, eventually returning through the umbilical arteries to the placenta for reoxygenation [22,23,24]. Following birth, the neonate’s first breath causes the lungs to expand with air, resulting in a significant decrease in pulmonary vascular resistance. This pressure differential forces the septum primum against the septum secundum, functionally closing the foramen ovale. The interatrial septum, as a barrier between the right and the left atria, keeps them separate. In this way, the blood from these two chambers doesn’t mix with each other [19,20,21].

ASD describes a condition where the right and left atria don’t close completely and remain open even after birth. There are four types of atrial septal defects depending on the location: ostium secundum defect (accounting for the majority of ASD cases), ostium primum defect (constituting 10% of ASD cases)—frequently associated with trisomy 21, sinus venosus defect, and coronary sinus defect [1,5,7,25,26]. Among them, the ostium secundum defect is the most common, serving as a window between the two atria that should not exist after birth. This defect permits abnormal left-to-right shunting of blood due to postnatal pressure gradients between the atria [1,5,7,25].

Clinically, ASDs may be identified during routine physical examination. In paediatric patients, characteristic auscultatory findings include a grade 2 to 3/6 midsystolic ejection murmur and a widely split, fixed second heart sound (S2) best heard at the upper left sternal border [27,28]. These signs, however, may be absent in neonates and young infants, particularly in cases of small defects [5,29].

### 2.2. Genetic Predispositions to ASD

Although most atrial septal defects (ASDs) occur sporadically, a subset arises in the context of autosomal dominant genetic syndromes and may co-occur with other congenital anomalies. For instance, Holt–Oram is associated with mutations in the TBX5 gene located on chromosome 12q24.1. Familial forms of ASD accompanied by progressive atrioventricular conduction disturbances have been linked to mutations or haploinsufficiency of the NKX2.5 gene on chromosome 5. In contrast, familial ASDs without associated conduction abnormalities may be attributed to mutations in GATA4. Additionally, abnormalities in other genes involved in atrial septal development, such as MYH6 and TBX20, have been implicated in the pathogenesis of interatrial septal defects [7,30,31,32]. Environmental factors, particularly prenatal exposure to teratogens, also contribute to the development of ASDs, as exemplified by cases associated with fetal alcohol syndrome [32].

## 3. Pathophysiology and Classification of ASD

### 3.1. Types of ASD

The majority of ASD cases arise sporadically, without a clearly defined etiology. However, those defects are often observed in various genetic syndromes, such as Down syndrome (~80%), Holt–Oram (~65%), Ellis–Van Creveld (~60%), and Noonan syndrome (~20%). Additionally, certain diseases of a mother, like diabetes, have been correlated with a heightened likelihood of ASD in offspring. Similarly, some environmental exposures, such as the usage of tobacco or antidepressant medications and alcohol intake during pregnancy, also disturb organogenesis [10].

**Figure 1:**
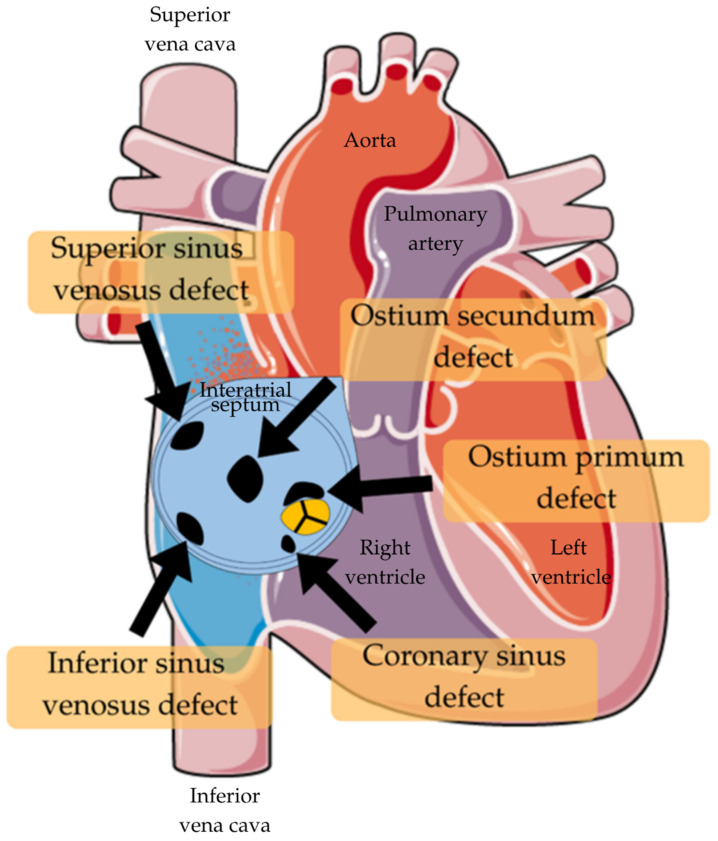
Types of ASD [35].

There are four types of ASD (Table 1; Figure 1). The most prevalent is ostium secundum, which accounts for around 75% of cases. It’s the defect occurring in the region of the fossa ovalis [33]. Another variant occurs when the septum primum fails to merge with the endocardial cushions found between the atria and ventricles. This results in a usually extensive defect at the atrial septum’s base. It is called ostium primum and is considered to be a part of an atrioventricular septal defect (AVSD) and is associated with atrioventricular valve anomalies. Moreover, this type of ASD induces more negative long-term effects than ostium secundum, no matter its diameter [34]. There is also a possibility of this abnormality existing near the junction of the superior vena cava (SVC) or inferior vena cava (IVC) and the right atrium. It is often linked to abnormal pulmonary venous return. The last type is classified as the coronary sinus ASD, because of its location, and is the rarest form of them all [33].

### 3.2. Left-to-Right Shunt Dynamics

**Figure 2:**
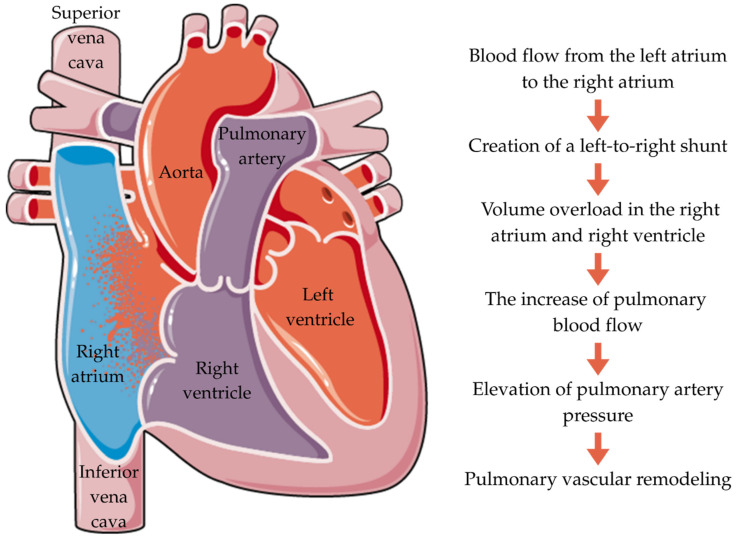
Pathophysiology of ASD.

ASD typically leads to a left-to-right (L-R) shunt (Figure 2). Blood flow through a tiny ASD is regulated by its size and relative atrial pressures, which are influenced by the ventricles. Large ASDs have roughly similar atrial pressures, and the ratio of the left ventricle (LV) and right ventricle (RV) compliances determines the shunt. Typically, the RV has higher compliance than the LV, causing an L-R shunt across the ASD. The amount of the shunt is determined by the difference in compliance between the RV and LV. LV compliance remains steady for the first 20–30 years of life. As people age, their blood pressure rises due to decreased arteriolar flexibility and increased systemic vascular resistance. This leads to increased energy use by the LV [36].

Laplace’s law predicts that greater afterload leads to LV hypertrophy. The LV’s compliance declines, leading to increased left atrium (LA) pressure and L-R shunt. The shunt causes right-sided volume overload. The expanded volume of the shunt causes enlargement of the pulmonary arteries, pulmonary vascular bed, right atrium (RA), and RV. The RV continues to widen until it fails, causing tricuspid regurgitation (TR). Initially, an increase in flow causes a reversible increase in pulmonary arterial pressure. However, with the hypertrophy of the wall of the pulmonary arterioles, pulmonary arterial hypertension (PAH) becomes severe and permanent [36]. Thus, the presence of an L-R shunt directly leads to a pulmonary vascular disease [37].

### 3.3. Hemodynamic Consequences

The severity of hemodynamic consequences depends mostly on the defect area and the size of the pulmonary vascular resistance. The persistent left-to-right shunt leads to an increase in blood volume and oxygenation in the chambers of the right heart and the pulmonary trunk. The greater amount of blood flow through the right heart causes volume overload and the enlargement of the right atrium and right ventricle, resulting in the appearance of specific symptoms. The decrease in diastolic volume of the left ventricle develops with age and intensifies clinical manifestations [38]. Moreover, ASD is associated with the risk of left ventricular dysfunction, caused mostly by diastolic function disorders [39]. Furthermore, in cases of ostium primum, there is a great number of patients with additional valve defects, which leads to increased mortality, heart block, heart failure, and advanced pulmonary vascular disease [40].

The enlargement of heart chambers can lead to the development of atrial arrhythmias, such as atrial fibrillation and atrial flutter, especially in older patients [41]. In some cases, where right atrial pressure exceeds left atrial pressure, a right-to-left shunt may occur. This allows the thrombi’s passage from the venous circulation to the arterial circulation, leading to a paradoxical embolism or an ischemic stroke. This can be particularly dangerous for patients with additional risk factors, such as antiphospholipid syndrome or the presence of venous thrombi [42].

The chronic increase in blood flow to the pulmonary vessels can lead to elevated pulmonary artery pressure, exceeding the normal value of mean pulmonary arterial pressure (mPAP) (>0 mmHg). The correlation between factors, such as age and the size of ASD, and PAH is low. This complication can be found in 10–20% of cases. Some patients don’t develop PAH despite a large ASD, whereas others may develop early symptoms due to a small ASD [43].

The aforementioned hemodynamic alterations can lead to various clinical presentations. They are mostly asymptomatic in early life. As the volume overload and pressure increase, patients may develop heart failure and experience exercise intolerance, dyspnea, and palpitations [10]. In order to prevent or minimize ASD’s manifestations, treatment methods were invented. The most commonly used is percutaneous closure, which is minimally invasive, but not suitable for large defects [44]. The other option is a surgical repair, used mainly in cases with sinus venosus and primum ASDs [45].

## 4. ASD and Pulmonary Hypertension

### 4.1. Definition

Pediatric pulmonary hypertension is defined as an increase in mean pulmonary arterial pressure (mPAP) ≥ 20 mmHg at rest, in children older than 3 months, measured by right heart catheterization [37]. Pediatric PH is classified according to the 6th World Symposium on Pulmonary Hypertension (WSPH) guidelines, with five major groups with similar pathophysiological mechanisms, clinical presentation, and management. In the context of ASD, PH generally falls under group 1, which is pulmonary arterial hypertension (PAH), including PAH associated with congenital heart disease (PAH-CHD), such as ASD [46].

### 4.2. Mechanism of Disease Progression

In children with ASD, chronic left-to-right shunting occurs and results in increased blood flow through the pulmonary circulation. Initially, the pulmonary vessels are able to adapt to the increased volume; however, sustained overload leads to pulmonary vascular remodeling. Subsequently, these pathological changes in the walls of the pulmonary arteries increase pulmonary vascular resistance (PVR). As PVR increases, pulmonary artery pressure also increases to overcome the resistance and maintain pulmonary blood flow [47]. In some cases, when pulmonary vascular resistance exceeds systemic vascular resistance, the direction of leakage may reverse to a right-to-left shunt. This could result in the development of cyanosis—Eisenmenger syndrome, indicating irreversible changes in the pulmonary vessels [17,48].

### 4.3. Comparative Risk of PAH in Ostium Primum and Secundum ASDs

Ostium primum atrial septal defects are associated with a higher incidence, earlier onset, and more severe progression of PAH compared with ostium secundum defects [49]. Moreover, in ostium primum ASDs, the left-to-right shunt frequently manifests in association with atrioventricular valve regurgitation, which promotes increased pulmonary blood flow and earlier pulmonary vascular lesions. In order to prevent further disease progression and complications, surgical intervention is required [5,50,51].

In contrast, ostium secundum defects, despite being the most prevalent type of ASD, lead to PAH less frequently and are typically characterized by a slow rate of progression with a more stable clinical course. Most small and asymptomatic defects warrant observation only [52], whereas closure is generally performed in the presence of hemodynamically significant shunts rather than due to mild pulmonary pressure elevations [5].

### 4.4. Eisenmenger Syndrome

Eisenmenger syndrome (ES) is a relatively rare multisystem disorder that represents a severe form of PH associated with uncorrected congenital heart disease (e.g., atrial septal defect); however, it is still prevalent and requires a multidisciplinary approach [18,53,54]. In children with ASD, this late complication usually manifests after many years of uncorrected shunting in adolescence or adulthood, and leads to central cyanosis, secondary erythrocytosis, and various multiorgan complications that significantly impair the quality of life [17,53,54]. Once Eisenmenger syndrome develops, ASD closure is contraindicated, and management is primarily symptomatic, involving a pulmonary pressure reduction to maintain stability. The disease-targeting therapies (DTT) include endothelin receptor antagonists as a first-line treatment and, additionally, phosphodiesterase-5 inhibitors, and are associated with a better survival rate; however, the rate is still unsatisfactorily low [17,18]. There is therefore a need for further research to guide management to improve survival prospects.

## 5. Clinical Presentation and Diagnosis

### 5.1. Symptoms

ASD is a heart defect that is usually not diagnosed until adulthood, often being discovered incidentally. When the shunt is large, symptoms such as arrhythmia, pulmonary hypertension, and heart murmurs may occur. Furthermore, children with ASD manifest suboptimal weight progression, increased respiratory rate, limited physical endurance, and are more prone to pneumonia [1].

Studies clearly indicate that the presence of ASD is associated with increased mortality (MRR 1.72), which rises with the patient’s age [16,55]. This dependency is influenced by the frequent lack of symptoms in the first decades of life, and when symptoms do appear, they often occur in conjunction with pulmonary hypertension or congestive heart failure [16]. It has also been observed that patients with ASD have an increased risk of developing atrial fibrillation (AF) later in life, even after defect closure [56].

According to Muroke et al., in patients with ASD, deaths from stroke and ischemic heart disease occur more frequently. However, female patients with ASD exhibit lower mortality than male patients (MRR 0.66). Patients who undergo ASD closure before the age of 30 show mortality rates similar to those of the control cohort. Furthermore, patients with transcatheter-closed defects have significantly lower mortality than those who undergo surgical closure (MRR 0.37) [55]. These findings underscore the importance of long-term cardiology follow-up in this group of patients.

### 5.2. Diagnostic Tests

Tests such as Transthoracic Echocardiography (TTE), Transesophageal Echocardiography (TEE), Cardiovascular Magnetic Resonance Imaging (CMR), Cardiovascular Computed Tomography (CCT), or Cardiac Catheterization are used during diagnosis to assess the morphology of the defect, determine its clinical significance, evaluate the possibility of percutaneous closure, and exclude any concomitant intracardiac defects. However, the most important method is ECG, which helps assess right ventricular volume overload and is essential for quantifying the defect’s hemodynamic relevance [57].

TTE allows for the identification of the type of ASD, assessment of defect size, determination of shunt direction, and preliminary qualification of patients for percutaneous ASD closure. TEE, on the other hand, allows for definitive evaluation regarding surgical intervention. Furthermore, three-dimensional TEE (3D TEE) enables even more detailed measurement of defect morphology, providing an alternative to balloon sizing, which carries certain procedural risks [58]. In the context of pulmonary hypertension, right heart catheterization is the most accurate method for evaluating pulmonary artery pressure (PAP) in patients with ASD [8].

As artificial intelligence continues to advance, new methods for detecting ASD have emerged. One such approach is AI-assisted automated auscultation; however, this method remains limited, as it does not allow for precise identification of the defect type, size, or hemodynamic significance (i.e., the direction of the shunt). Another emerging technique involves the analysis of echocardiographic images using deep learning algorithms. While promising, this method is still under investigation, as accurate diagnosis of congenital heart disease (CHD) critically depends on standardized echocardiographic view recognition [59].

The most effective results to date have been achieved using color Doppler echocardiographic images in combination with convolutional neural networks (CNNs). Diagnostic accuracy is further enhanced by incorporating multiple acoustic windows—specifically subxiphoid, apical, and parasternal approaches—as well as multiple echocardiographic views. Recent findings indicate that these systems are capable of reliably detecting ASD, marking a significant advancement toward the integration of AI into standard CHD diagnostic practices [59].

### 5.3. Clinical Presentation

Transthoracic echocardiography often reveals diastolic septal flattening and a D-shaped left ventricle, indicative of right ventricular volume overload. This mechanical shift limits left ventricular filling due to compression from the dilated right ventricle. As a result, blood flow is redirected from the left to the right atrium, reducing effective preload and cardiac output [16].

Despite a lower end-diastolic volume, left ventricular pressure may remain normal or elevated, reflecting impaired compliance. Studies have demonstrated altered diastolic pressure-volume relationships in ASD patients [60]. While closure of the defect normalizes external loading conditions, inner factors such as diastolic stiffness or relaxation abnormalities may persist. These are particularly relevant in older adults, who are at higher risk of transient heart failure post-closure due to age-related myocardial stiffening. In contrast, children generally maintain preserved diastolic function and tolerate closure better [16].

Right ventricular volume overload reduces left ventricular preload and myofiber stretching. Consequently, stroke volume decreases, consistent with Starling’s law. Chronic, significant shunting results in right-sided heart volume overload, which can lead to myocardial damage. This is supported by elevated levels of highly sensitive cardiac troponin I in ASD patients compared to controls. The enlargement of right-sided chambers increases myocardial oxygen demand, causing relative myocardial hypoperfusion. Elevated levels of angiotensin II and catecholamines may contribute to myocardial injury through cell necrosis and apoptosis [16].

The amino-terminal pro-collagen type III peptide, a marker of collagen synthesis, is elevated in heart failure and correlates with the pulmonary-to-systemic blood flow ratio in ASD patients. It may also reflect myocardial stiffness and remodeling, which are linked to diastolic heart failure [16]. Pulmonary hypertension is observed in approximately 6–35% of patients with an atrial septal defect [12]. It is associated with increased mortality and significant functional impairment. Furthermore, ASD is more frequently found in preterm infants and in children with chromosomal abnormalities [61].

## 6. Managing Atrial Septal Defect and Pulmonary Hypertension in Children

In this section, we discuss how to care for children with atrial septal defects (ASDs) and how to treat PH linked to these defects with medication [11,12]. For secundum ASDs that do not cause issues for the heart or raise pressure in the pulmonary artery, it is common to take a cautious approach and wait, especially if the children show no symptoms [14]. Important factors to consider include the chance of the defect closing on its own, the optimal time to intervene, and making decisions tailored to each child based on imaging and clinical assessments [11,62]. In the context of ASDs (Atrial Septal Defects), managing PH poses a challenge due to its complex pathophysiology; it often necessitates a strategy of treating first and then assessing for repair to lower pulmonary vascular resistance (PVR) and evaluate the feasibility of surgery [13,14,63]. These paragraphs outline the existing knowledge on monitoring practices, interventions, and ongoing care to aid in making informed clinical decisions.

### 6.1. Watchful Waiting for Minor ASDs

ASDs that do not result in strain on the right side of the heart or increased pressure in the lungs are usually managed with a cautious approach, particularly in children without symptoms [14]. Small defects measuring less than 5 mm often close naturally before a child turns two years old [15,62,64]. Regular echocardiograms play a key role in tracking how these defects evolve and identifying any changes to the right side of the heart or an increase in shunting flow [63]. Premature babies and children with conditions like Down syndrome or heart and lung issues are less likely to experience spontaneous closure of certain heart defects compared to others without these challenges, suggesting that surgical intervention might be needed sooner for some with lung diseases like bronchopulmonary dysplasia, even for small ASDs [12,13]. Nonetheless, there is evidence indicating that in some cases, it may be better to wait until lung function improves before considering closure procedures [62,64].

The optimal time to close an ASD in children is still being studied by experts in medicine and child healthcare services to determine the most suitable age for a closure procedure that benefits the child’s health and well-being in the long term, per current medical standards and practices generally followed worldwide [14,15]. Typically, doctors plan for ASD closure between ages two and five as per medical protocol; however, early intervention may be necessary if signs of right ventricular dilation are detected or if the defect size exceeds 8 mm, based on medical guidelines and recommendations [11]. When deciding when to proceed with ASD closure surgery or intervention, it is crucial to evaluate each patient individually by considering results from diagnostic imaging tests, assessments of heart function, and specific risk factors unique to each patient that might influence the timing of the procedure, in close consultation with specialized healthcare professionals involved in their care journey [11,14,62,63].

### 6.2. When and How to Close ASDs

Patients with ASDs measuring less than 5 mm frequently undergo spontaneous closure of the defect within the first year of life. It is hypothesized that these defects represent a stretched patent foramen ovale rather than true secundum atrial septal defects (ASDs). It has been established that Ostium primum atrial septal defects (ASDs) and sinus venosus ASDs do not close spontaneously. Defects larger than 8 mm generally necessitate medical or surgical intervention for closure [14,65,66]. Sinus venosus, ostium primum, and coronary sinus septal defects invariably require surgical repair [65,67]. In instances where there is a shift in blood flow from the left side to the right side of the heart (with a ratio greater than 1.5:1), along with an enlarged right heart and symptoms like difficulty breathing, frequent lung infections, irregular heartbeats, or pulmonary hypertension, closure may be required promptly if pulmonary vascular disease worsens or cardiac function is compromised [7,11,68].

Significant advancements have been made since the first atrial septal defect surgery in 1948, with over 70 years of experience enabling procedures to be performed with minimal mortality or morbidity. The advent of contemporary minimal access techniques, such as video-assisted thoracoscopy and robotic surgery, has engendered a paradigm shift in the domain of surgical practice. These methodologies have been shown to be both safe and reproducible, while concomitantly delivering enhanced cosmetic outcomes and expedited recovery periods [67]. In the case of ASDs necessitating closure, a range of options exists, encompassing both percutaneous and surgical interventions. Percutaneous transcatheter closure poses less risk for the patient but is only suitable for closing ostium secundum defects [5]. The limitations of transcatheter closure predominantly pertain to the dimensions of the defects (in cases where the defect is too large) or the physical dimensions of the infant (in cases where the infant is too small). The following relative contraindications to device closure of secundum defects have been identified:

- Very large size (diameter greater than 36–40 mm);
- Inadequate margins for device anchorage;
- Potential device interference with atrioventricular valve function;
- Potential obstruction of systemic or pulmonary venous drainage [67,69,70].

Newer options, like the Carag bioresorbable occluder, aim to lower the risk of long-term complications by enhancing tissue integration; however, detailed long-term data on their effectiveness are still scarce [62]. Ostium primum atrial septal defects (ASDs) are characterised by a deficiency of septal tissue between the atrioventricular valves. Surgical repair is imperative and involves the direct suturing of a patch to valve tissue while preserving the conduction system and underlying ventricular septum. Concurrent mitral valve cleft repair is frequently undertaken to enhance valve function. Surgical correction is typically achieved by means of a patch closure of the septal defect, with attention being paid to the left atrioventricular valve cleft in order to ensure functional integrity [5,71].

**Figure 3:**
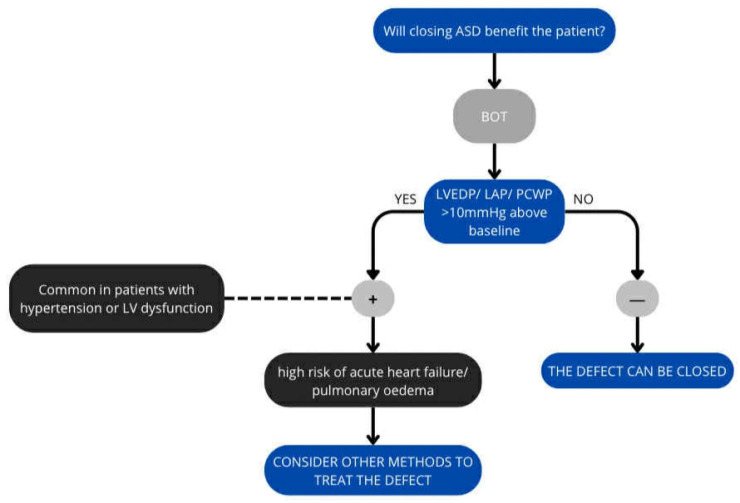
Algorithm for ASD closure.

Given the risks associated with the operation, the benefits of surgery must be assessed before making a final decision on ASD closure (Figure 3). The balloon occlusion test is used for this purpose. The balloon occlusion test (BOT) in heart failure patients, particularly those with an atrial septal defect, is an invasive procedure to predict the risk of acute left ventricular failure after permanent closure [6,72].

The ASD is temporarily closed by a balloon (sizing balloon used to select device size)—the balloon is placed at the leak for 10–20 min to stop blood flow, during which time the PCWP, LAP or LVEDP is measured. The test reveals how the left ventricle and pulmonary circulation respond to increased pressure [72,73,74].

Any significant augmentation (>10 mmHg from the baseline value) of LAP or LVEDP during balloon occlusion is considered a positive balloon occlusion test (Some authors point to the possibility of using PCWP values, but this is considered less accurate). A positive test result indicates a high risk of acute pulmonary oedema or heart failure, and the procedure should be abandoned. It also mandates further pharmacological intervention prior to closure or consideration of a fenestrated closure device [72,73,75].

Transcatheter closure represents a prevalent technique employed in the treatment of secundum atrial septal defects (ASDs). In comparison with surgical interventions, it offers a less invasive approach, a more rapid recovery period, and a diminished physical and psychological impact. In the contemporary era, the transcatheter closure of secundum ASDs has become a prevalent procedure, with a success rate ranging from 85 to 90% [76]. In the context of secundum ASDs, a median sternotomy facilitates access to the harvesting of autologous pericardium for the purpose of achieving a tension-free repair. The initiation of cardiopulmonary bypass is followed by the achievement of diastolic arrest using cardioplegia. Intraoperative assessment is a crucial component of surgical planning, as it ensures the precise localization and correction of the malformation without compromising the integrity of surrounding structures, such as the tricuspid valve, coronary sinus, and atrioventricular node. Postoperative transesophageal echocardiography with a bubble study is a confirmatory procedure that provides a comprehensive evaluation of the integrity of the repair and the presence of any residual shunting [5].

In the case of an uncomplicated secundum defect, intracardiac repair is a relatively straightforward procedure. The right atrium can be opened in either a longitudinal or a transverse direction, and small atrial septal defects (ASDs) can be closed by direct suture. In the case of larger defects, the most effective course of action is often a patch repair. The conventional approach, involving a median sternotomy and central cannulation, has become remarkably safe, straightforward, and reproducible. Advancements in this field have focused on surgical access with the objective of reducing trauma and accelerating recovery [67].

Sinus venosus atrial septal defects (ASDs), which are frequently associated with anomalous pulmonary venous drainage, necessitate careful dissection in order to delineate pulmonary vein anatomy and to prevent injury to adjacent structures, such as the phrenic nerve. The methodology employed in such repairs is contingent upon the complexity of the defect and the anatomical variations present, with one-patch, two-patch, or Warden procedures being the standard techniques [5]. The Warden procedure, which involves the transection of the superior vena cava (SVC), its subsequent connection to the right atrial appendage, and the baffling of pulmonary venous return, has been shown to yield durable outcomes [77]. However, it is imperative that meticulous attention is paid to achieving a tension-free anastomosis in order to avoid complications such as SVC obstruction or sinus node dysfunction [5].

Given the proximity of this defect to the septum adjacent to the vena cava, partial anomalous pulmonary venous drainage is commonly present, typically involving the right upper and/or middle pulmonary veins draining anomalously to the atrium or SVC. In instances where anomalous pulmonary venous drainage is in close proximity to an ASD, the surgical intervention to address the anomaly is relatively uncomplicated and shares similarities with the repair of secundum ASDs. However, in more frequent instances, the anomalous pulmonary veins drain to the superior vena cava (SVC), necessitating either a two-patch or Warden technique [67]. The two-patch technique involves the utilisation of one patch for the closure of the ASD and another for the closure of the right atriotomy at the cavoatrial junction. This configuration is designed to prevent the development of stenosis in the superior vena cava (SVC) and the right atrium. The right atrial incision frequently extends onto the superior vena cava (SVC), which can result in sinoatrial node dysfunction [67,78].

Conversely, the Warden technique entails the division of the SVC above the anomalous pulmonary veins, followed by anastomosis to the right atrial appendage. The residual SVC, now receiving only pulmonary venous blood, is baffled to the ASD, thereby effectively separating the atria [67,78]. This approach avoids the need for a cavotrial incision, thereby reducing the risk of sinus node dysfunction. However, late SVC obstruction at the right atrial anastomosis has been reported, necessitating adequate resection of pectinate muscles within the right atrial appendage to mitigate this complication [67,79]. Modifications of the Warden technique, such as the use of the right atrium as a superior-based flap to the SVC with an anterior pericardial patch or the performance of a direct posterior SVC-to-right atrial anastomosis with an anterior pericardial patch, aim to minimise tension and stenosis, particularly in patients with high pulmonary vein entry to the SVC [67].

A follow-up echocardiogram should be performed 24 h after the procedure to rule out effusion. Subsequent echocardiograms should be performed after 1–3 months, then after one year, and then every 2–4 years [7,80].

### 6.3. Treating ASD and PAH

Pulmonary hypertension linked to ASDs arises from causes like higher blood flow in the lungs and preexisting lung vascular issues. Predicting whether pulmonary hypertension can be reversed is crucial in deciding the safety of closing an ASD [11,12,63]. The strategy of “treating before repairing” starts with using medications to lower PVR when it is high and reversible, such as endothelin receptor antagonists like bosentan, phosphodiesterase-5 inhibitors like sildenafil, and prostacyclin analogues [12,13]. Sometimes, combining these treatments may be required to improve heart and lung function before further action is taken [12,13,81]. A thorough assessment includes echocardiography, MRI, and cardiac catheterization with vasoreactivity testing to determine operability indicators, such as a PVR below 6 Wood units and a PVR/SVR ratio under 0.33, which are considered crucial criteria for determining operability levels. In challenging scenarios, like Lutembacher syndrome, a sequential strategy involving procedures such as balloon mitral valvotomy followed by ASD closure might be necessary [11].

The decision to close an ASD in the presence of PAH is complex and necessitates a multidimensional evaluation, and general recommendations cannot be made [11,82]. Nevertheless, it is primarily determined by the severity of pulmonary vascular disease, assessed by the Pulmonary Vascular Resistance (PVR). According to the 2022 ESC/ERS pulmonary hypertension guidelines, ASD closure is generally considered safe in patients with a PVR < 3 Wood Units (WU) [37]. In cases with moderately elevated PVR (3–5 WU), closure may be considered in specialised centres, particularly when pulmonary vasodilator therapy demonstrates favourable hemodynamic reversibility. Closure is contraindicated in patients with PVR ≥ 5 WU or fixed, non-reversible pulmonary vascular disease due to the risk of precipitating RV failure and Eisenmenger syndrome. In paediatric populations, early intervention is advocated to prevent the development of irreversible pulmonary vascular remodelling, whereas a ‘treat-and-repair’ strategy (initial pulmonary hypertension–targeted therapy followed by reassessment of suitability for defect closure) is typically reserved for borderline cases [37,83].

In cases of heart failure (HF), pharmacotherapy is recommended (Table 2). However, it acts as a support for causal treatment. It is advised to avoid aggressive volume changes, which may lead to increased or reduced systemic output, causing hemodynamic shifts. Monitoring of blood pressure (BP), heart rate (HR), renal function, electrolytes (K^+^), and clinical symptoms plays a key role in successful therapy.

### 6.4. Outlook and Long-Term Care

Lifelong, structured follow-up (Table 3) for patients with ASD is essential to monitor arrhythmias, progressive PH, and Eisenmenger syndrome. Timely identification and management of these complications is pivotal for optimizing long-term outcomes, as recommended by ESC guidelines. Follow-up intervals should be individualized, with annual or more frequent assessments in patients with elevated PH risk [83].

Children who receive ASD closure before irreversible changes occur in their hearts or rhythm issues generally have positive outcomes afterward. The size of the right side of the heart usually returns to normal within 6 to 12 months after the procedure, leading to improved exercise capacity and growth [62,64]. After the procedure, follow-up examinations are essential, including physical check-ups alongside electrocardiography and echocardiography at 1-month post-closure, followed by assessments at 6 months and 12 months, with subsequent yearly evaluations. For individuals with existing PH or complex anatomical issues, advanced imaging techniques or intermittent catheterization may be required for ongoing monitoring of their long-term condition [7,12].

Complications like device erosion or residual shunting may arise in patients with delayed closure or significant preoperative cardiac dilation, highlighting the need for monitoring due to potential risks [14,81]. Emerging technological advancements, such as absorbable occlusion tools and minimally invasive surgery methods, could lead to better outcomes in the future. Telehealth services and portable medical tests can improve healthcare access in areas with limited resources. Worldwide databases, like the International Quality Improvement Collaborative (IQIC), aim to standardize healthcare practices and ensure treatment accessibility for all [64].

## 7. Discussion

Despite growing knowledge about ASD and the mechanisms leading to pulmonary hypertension resulting from its presence, there is still no consensus on the timing and method of intervention after diagnosis. The classic indications for medical intervention—significant left-to-right shunting leading to right ventricular volume overload—do not apply to specific clinical situations, such as children with genetic syndromes or coexisting lung disease.

The indication for ASD closure is significant left-to-right shunting, which leads to enlargement of the right heart chambers as a result of volume overload. In the case of concomitant PAH, classification for surgery is more difficult, and data obtained during right heart catheterisation may be useful. PH in patients may limit exercise capacity and may result in heart failure, so it is a factor limiting closure [7]. The possibility of closure is limited by PAP and PVR. When, despite pharmacotherapy, PAP and PVR > 5 WU, this is a contraindication to ASD closure. It is difficult to identify patients with ASD and PH in whom closure would have a positive therapeutic effect. In children, changes associated with right ventricular overload occur gradually; hence, the most common decision is to postpone surgery until the child is 3–5 years old [7,61]. It is important to monitor the size of the defect, which may increase significantly with age. Unfortunately, there are no clear criteria for classifying children for surgery, so each case is considered individually [7].

However, there are also studies showing positive results from much earlier interventions. The authors of these studies emphasize that earlier closure of the defect prevents further damage to the pulmonary vessels. Early closure of ASD leads to a reduction in blood flow through the pulmonary vessels and, consequently, may lead to improved lung mechanics. This was noted by Tsuda et al., who observed an improvement in clinical condition and resolution of PHTN after intervention in patients under 2 years of age [85]. Wiegand et al. also observed improved hemodynamics after early intervention in infants with lung disease [86]. These results suggest that early surgery may be beneficial, especially in certain groups. Despite the promising results, it should be remembered that the trials involve small groups and the follow-up rarely exceeds a few years. The discussion should also refer to the results of the study by Lammers et al. conducted on an adult population (patients over 16 years of age) should also be mentioned in the discussion, which emphasizes that early intervention does not completely eliminate the risk of developing PHTN. The researchers observed the development of pulmonary hypertension in some patients despite successful closure of the ASD [87].

Some experts advocate a strategy of “watchful waiting,” emphasizing that closing an ASD with irreversible changes in the pulmonary vessels may be harmful. In their article, Jain et al. point to the risk of developing life-threatening acute pulmonary edema after intervention in patients with restrictive left ventricular (LV) physiology [11]. Furthermore, a study by Muroke et al. shows that patients who underwent percutaneous ASD closure are at greater risk of AF, migraine, and have an increased risk of atrioventricular conduction disorders and ventricular fibrillation/tachycardia. However, the results of this study may be less relevant to the pediatric population, as the group of patients in this study was heterogeneous in terms of age, with patients under 18 accounting for approximately 32% [88].

Currently, there is a tendency to close increasingly larger cavities in increasingly younger patients, thanks to the possibilities offered by modern occluder devices and implants. We still have rather limited access to information about the long-term effects of using these devices. Long-term studies (>5 years) on occluder devices show excellent closure rates and low late complications; however, rare events such as device embolization, arrhythmia, new valve regurgitation, or mild residual shunts are documented. The advantages of using the new devices include: a reduction in the amount of permanent foreign material, a lower risk of further long-term complications, and better anatomical adaptation over time: as the child grows, the resorbable structure can provide a certain degree of ‘flexibility’, reducing the risk of pressure on adjacent structures. However, this is still a matter of speculation [89].

In summary, the decision to close ASD in pediatric patients must be personalized and based on an assessment of risk factors and the potential benefits of intervention. Further multicenter studies are needed, focusing on the long-term outcomes of intervention in the pediatric population and specific groups, leading to the development of evidence-based criteria for intervention to facilitate decision-making in everyday clinical practice.

## Figures and Tables

**Table 1:** Types of ASD [33].

Type of ASD	Developmental Defect	Location	Associated Features
Primum ASD	Incomplete formation of the inferior limb of the septum secundum.	Lower part of the atrial septum.	Often linked with a cleft anterior mitral leaflet; considered a partial atrioventricular septal defect (AVSD).
Secundum ASD	Defect in septum primum (fossa ovalis) or upper septum secundum.	Central part (fossa ovalis region).	May be single or fenestrated (multiple small defects).
Sinus Venosus ASD	Abnormal development near venous entry points; deficiency of tissue separating pulmonary veins and vena cava.	Near the superior vena cava and inferior vena cava.	Frequently associated with abnormal pulmonary venous return.
Coronary Sinus ASD	Unroofing or absence of the coronary sinus.	Area of the coronary sinus ostium.	Can lead to communication between the atria through the coronary sinus.

**Table 2:** Medications in heart failure [84].

Drug Type	Active Substances	Goal	Notes
Angiotensin Converting Enzyme (ACE) Inhibitors	Ramipril, Enalapril, Lisinopril	Reduction in afterload and halted LV remodelling.	Use in case of LV dysfunction or hypertension; caution with excessive afterload reduction.
Angiotensin Receptor Blockers (ARBs)	Losartan, Valsartan	Alternative to ACE-I.	Use when ACE-I is not tolerated.
Angiotensin Receptor Neprilysin Inhibitors (ARNI)	Sacubitril + Valsartan	Newer alternative to ACE-I/ARB.	For HFrEF or post-closure LV dysfunction, avoid if unstable BP.
Beta-blockers	Bisoprolol, Carvedilol, Metoprolol succinate	HR reduction, survival improvement, tachycardia prevention.	Start low in low-output or preload-dependent ASD; avoid bradycardia.
Mineralocorticoid Receptor Antagonists	Spironolactone, Eplerenone	Halted remodelling.	Use in chronic HF phenotype; avoid if K^+^ > 5 or eGFR < 30.
SGLT-2 Inhibitors	Empagliflozin, Dapagliflozin	Reduced hospitalizations and mortality.	Adjust if eGFR < 30; watch for dehydration.
Loop Diuretics	Furosemide, Torasemide, Bumetanide	Congestion and fluid overload decompression.	Avoid overdiuresis.
If-channel Inhibitor	Ivabradine	HR reduction in sinus rhythm when HR ≥ 70 bpm despite a beta-blocker.	Only if sinus rhythm and symptomatic; avoid in AF.
Hydralazine + ISDN	Hydralazine + Isosorbide dinitrate	Alternative/add-on vasodilator in intolerance to RAAS blockers.	Use with caution in ASD (hemodynamic shifts); watch BP.
Digoxin	Digoxin	Rate control in AF, mild inotropy in LV dysfunction.	High toxicity risk.

**Table 3:** Components of monitoring.

Monitoring Domain	Key Assessment Parameters
Clinical Evaluation	Exercise tolerance, dyspnea, syncope, palpitations
Cardiac Rhythm	ECG, Holter monitoring
Echocardiography	RV size and function, Tricuspid Regurgitation (TR) velocity, residual shunts
Radiology	Cardiac MRI
Biomarkers	NT-proBNP
Functional Testing	CPET, 6-min walk test
Hemodynamic Assessment	Right Heart Catheterization (RHC)
Hematology	Oxygen saturation (SpO_2_), Complete Blood Count (CBC)
